# All I Want for Christmas Is a Loo: Visualizations of Sex and Gender on Toilet Doors

**DOI:** 10.32872/cpe.16159

**Published:** 2024-12-20

**Authors:** Judith Rosmalen, Ilona Plug, Aranka Ballering

**Affiliations:** 1 Department of Psychiatry, University Medical Center Groningen, University of Groningen, Groningen, the Netherlands; 2 Department of Internal Medicine, University Medical Center Groningen, University of Groningen, Groningen, the Netherlands; 3 Department of Communication and Cognition, Tilburg School of Humanities and Digital Sciences, University of Tilburg, Tilburg, the Netherlands

## Preface

Ah, Christmas Eve. A time for carols, cozy fires, and, of course, culinary excellence. You’ve secured a reservation at one of your city’s best restaurants, ready to pamper your taste buds with gastronomic delights, until it all takes an unexpected turn. You want to run from the table and not because of your eating companion’s endless debates. Rather, your gut is staging a revolt, hinting at an encore performance of your prawn appetizer. Channeling your inner Olympic sprinting champion, you run to the restroom, breaking records and social decorum alike. And now, in front of the restroom doors, you face the ultimate dilemma: which door to choose? While your gut is rumbling along with Mariah Carey’s song playing in the background, you can only think: “All I want for Christmas is a clue…”

We are all confronted with the importance of a nuanced understanding of both sex and gender when facing a toilet door in dire times, but this day-to-day realization does not always find its way into the realm of health research (Ballering et al., 2023). Although health-related research is becoming increasingly sex-sensitive (i.e., attentive towards the biology of intersex, male, and female bodies), sensitivity towards gender (i.e., attentiveness towards psychosocial factors related to being a woman, man, or non-binary identity) in research remains scarce (Ballering et al., 2024). This is problematic, as it hampers the validity of conclusions drawn from these studies, as recent studies show that sex and gender independently affect health outcomes (Ballering et al., 2020; Mauvais-Jarvis et al., 2020).

Multiple resources are available to increase sex and gender-sensitivity in research, with the SAGER guidelines being most prominent (Heidari et al., 2016). However, we consider one key resource to be underused in raising gender-sensitivity in research: toilets. After all, we all choose on a daily basis which toilet door to open. This sensitivity to (the difference between) sex and gender in daily life, is ought to find its way to health research. Therefore, this study identified visual loo clues on toilet doors that guide the decision on which toilet to enter. We thematically analyzed, and subsequently quantified, whether these clues are related to sex or gender. We aim to sharpen the reader’s understanding of sex and gender, thus facilitating increased sex and gender sensitivity in research. Also, this will aid readers so that the next time they find themselves in front of those puzzling restroom doors, they confidently choose the clue for the right loo without breaking a sweat (or a sprinting record).

## Method

### Data Collection

We collected pictures of international toilet doors in the period of 2020 to 2022. Initially, we visited and photographed as many toilet doors as possible whenever we were outside of our homes. We did not seek permission from an ethical committee, nor from the owners of the toilet doors. Consequently, we had to endure confused looks of others while we were taking these pictures – ah, the things we do in the name of science. We also searched online using a search string in Google containing commonly used Dutch and English variants of the terms ‘toilet door(s)’, ‘toilet door sticker(s)’, toilet icon(s)’ and ‘toilet entrance’. Duplicates were defined as different doors having exactly the same visual clues; these were excluded from the analyses. All raw visual data are available from OSF (Rosmalen et al., 2024S-a).

### Coding of Visual Decision Clues as Related to Sex and/or Gender

Each set of toilet doors was independently analyzed by three female coders (mean age 35 [*SD* = 13.9]). The coders identified all visual elements on a given set of toilet doors that would aid the decision of which door to open in times of need. In some cases, predominantly for gender-neutral or gender-inclusive toilet doors, there was only one door to assess. Open coding of visual elements was used, followed by a thematic analysis. Consensus discussion resulted in a final definition of the visual clues per toilet door.

Visual clues were considered neutral, if these did not consistently represent either sex or gender, or as referring to either sex or gender. The three coders extensively discussed all themes in order to reach consensus on their categorization as neutral, sex, or gender clues. Interrater agreement was assessed by means of free-marginal multi-rater kappa using the Online Kappa Calculator (http://justusrandolph.net/kappa/). The overall agreement between the three raters was 85.5%; the free-marginal kappa was 0.78 (95% CI [0.61, 0.95]).

### Analysis

MS Office Excel was used to analyze the total number of clues and per set of doors.

## Results

### Study Sample

Appendix 1 in the Supplementary Materials shows the PRISMA statement. Our final study sample included 97 sets of toilet doors. Of these doors, six were sex-or-gender-neutral (i.e., not specifically related to women or men), unisex (i.e., specifically related to both female and male individuals) or even universal with visual clues that moved beyond sex and gender, as these depicted aged people, tattooed people, queer people, the Grim Reaper, people with various religions, aliens, fairies and mermaids.

### Thematic Analysis

On the 97 doors, we identified a total of 260 visual decision clues that were categorized in 23 themes. The minimum and maximum number of clues per door were 1 and 8, with a median of 2 (IQR 1-4). The 23 themes were subdivided into neutral (*n* = 2), or related to sex (*n* = 9) or gender (*n* = 12). The identified visual themes and concomitant frequencies can be found in Appendix 2.

Of the 260 clues, 52.7% were gender-related and 30.0% were sex-related. The remaining 17.3% included text and icons that were categorized as neutral. Sex clues were found on 54.1% of all doors, gender clues on 72.4%.

### Neutrality and Naughty Nomenclature

Two themes, namely text elements and icons, were coded as neutral, since these did not consistently represent either sex or gender. Textual sex clues included variants of ‘*Male-Female*’ or synonyms in different languages (e.g., ‘*Miehet-Naiset’*, ‘*Femei-Barbati’* or ‘*Vrouwtjes-Mannetjes’*), or in different species (e.g., ‘*Bulls-Cows’*). We also categorized the text element ‘*Sausage-Eggs’* as a sex clue, assuming that decision clues on toilet doors are not reflecting food preferences. Gendered textual clues include names (‘*Adam-Eve’*), cognitions (‘*Shopping-Football’*), communication (‘*Bla-Blablablabla’*), and assumed capabilities (‘*Men to the left, because women are always right’*)^1^1Clearly, the three coders, who identify as women, fully agree with the assumptions made on this toilet door..

**Figure 1:**
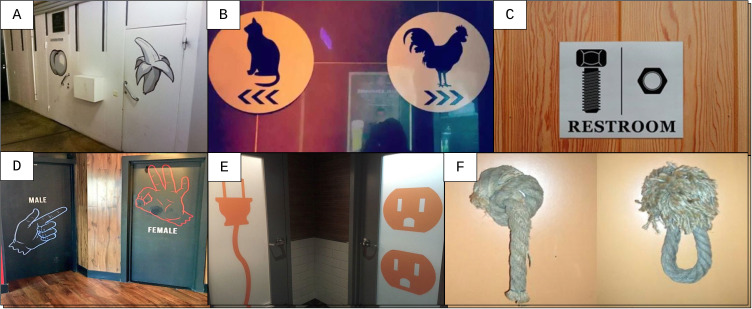
Toilet Doors With Icons

Icons predominantly included symbolic representations of the male and female reproductive organs (i.e., sex; Figure 1). Discussion was required for the doors in Figure 1B that contain a sophisticated symbol of the words used; an *N* = 1 pilot test in a young man suggested that these clues on the toilet door would take up too much time than preferred. One of the authors did not understand these doors either until the consensus discussion; as a linguist she interpreted these doors as representing gendered behavior: cocky (i.e., men-like) and catty (i.e., women-like).

### Peeing With Precision and the Angle of Male Confidence

**Figure 2:**
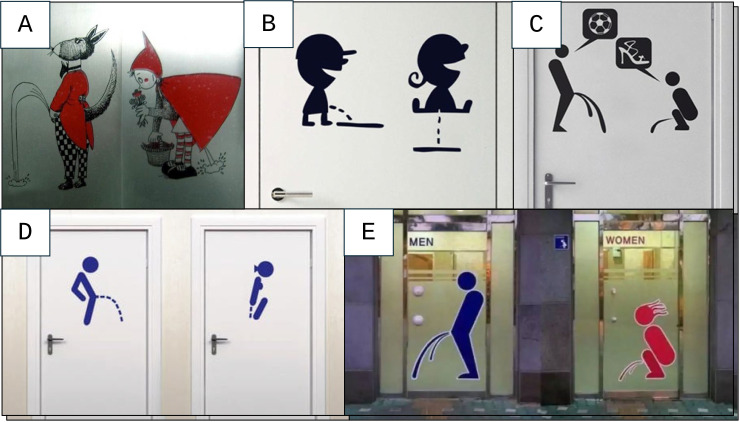
Urinary Streams

Some visual clues required some more extensive discussion. An example hereof is the urinary stream theme (Figure 2). We initially assumed that the urinary stream was a sex clue, since it would be related to anatomical characteristics of men and women. However, it appeared more complex. We assessed the initial angle of the male urinary stream, based on the Visual Prostate Symptom Score (VPSS) (Stothers et al., 2017). Most male icons in Figure 2 depicted an angle that exceeds the highest possible score on the VPSS, namely a strong horizontal or even sky-facing stream. Potentially, this indicates male (over)confidence, which would be gender-related. A validated female equivalent of the VPSS is unfortunately not known to the authors. However, we noted a women-specific aspect that might influence a urinary stream: the women sit, bend and even jump while peeing (Figure 2). We hypothesize that this is due to the female practice of urinating without trying to touch the public toilet seat, which may be related to gender.

### A Case of the Jingle Balls

**Figure 3:**
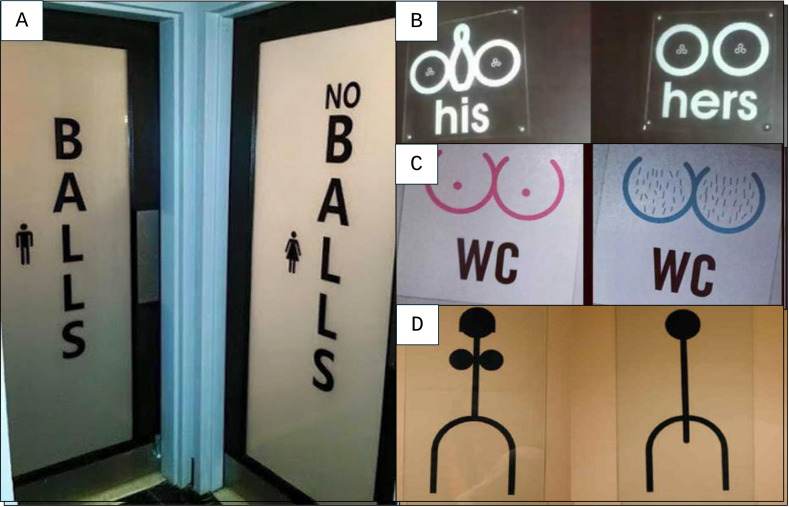
Toilet Doors With Balls

A second example that required somewhat more extensive discussion on the sex versus gender dimension of visual clues on toilet doors is provided in Figure 3. We initially assumed that the text element ‘*Balls-No balls’* in Figure 3A referred to sex (or potentially to how to decorate a Christmas tree). However, when studying doors with more direct graphical representations of balls, our interpretation appeared to be incorrect: Figure 3B shows similar balls for him and her, Figure 3C shows similar numbers of balls but qualitative differences, and Figure 3D suggests that no balls refers to men, which contrasts with Figure 3A. Since all graphical representations show that women have at least as many, or even more, balls than men, we assume that ‘*Balls-No balls*’ in Figure 3A refers to feminine gender and masculine gender respectively, in which case we -as three women- would like to point out that the owner of the doors misplaced the male/female icons.

## Discussion

Our study found that loo clues are primarily based on gender, as 52.7% of the clues depicted on toilet doors related to gender, while 30.0% related to sex. Twelve of the 23 identified themes related to gender and nine related to sex. Clues related to gender were found on 72.4% of the included toilet doors and sex clues on 54.1%.

Before interpreting our results, the following limitations need to be taken into account. First of all, data collection was performed by three women. We assume this has not influenced the offline search strategy, since we collected visual clues outside of the restrooms. One could argue that the female-oriented online search histories would have influenced the online search results, but the searches were done on computers that were also used by men. Secondly, selection bias may have occurred: it is likely that either extremely gendered or sexed toilet doors that were considered funny or interesting enough to be put online were found via our online searches. Lastly, the embodiment of gender, and what is considered feminine and masculine, depends strongly on time, place, and culture potentially affecting the interpretation of loo clues.

To the best of our knowledge, little to no previous studies have systematically assessed and quantified the clues present on toilet doors with regards to differentiating sex and gender (Iio, 2023). It has been previously argued that many toilet signs have a symbolic function (Iio, 2019). In our study, the majority of signage does not depict the toilet itself (only 4 clues) or the activity of using the facility (7 clues related to urinary stream and 10 to urinating position, often combined). Therefore, the signage of toilets frequently involves learned behavior and culturally accepted conventions that tie closely with gendered segregation of men and women, and use of toilet facilities (Ciochetto, 2003).

Previous research argues that explicitly showing the act of urinating or defecating itself is seldom reflected in toilet signage. Potentially, this may be culturally inappropriate (Ciochetto, 2003). However, our results contrast with this view. Especially with regards to the VPSS, the results also suggest that toilets are not very inclusive for men with prostate problems. This may be attributed to the stoicism related to masculinity, which results in men not easily admitting physical complaints or bodily disturbances (Ballering et al., 2021; MacLean et al., 2010). Toilet signage involving men with prostate problems may not be attractive for men without problems.

We conclude that current toilet signage predominantly uses gender clues to indicate who should enter. Further studies could investigate the amount of time it takes to make a decision based on sex and gender clues, in relation to attitudes towards sex and gender characteristics. As a practical implication, we suggest for all to take a second look at the loo clues and try to identify the sex and/or gender clues before entering a toilet, if the urgency allows the time investment^2^2If the urgency is relatively low, please consider snapping pictures of interesting toilet doors and send these to the corresponding author. Especially doors with Christmas trees, Santa Claus, and reindeers are very welcome.. This facilitates a learning experience about the difference between sex and gender and may subsequently increase sex and gender sensitivity in research as well. But, perhaps more importantly, you can now enjoy your holiday dinner in peace, knowing that the only dilemma left to solve this Christmas is whether to indulge in another slice of pie—without a hint of restroom anxiety in sight.

## Supplementary Materials

The Supplementary Materials contain the following items:

- All raw visual data (Rosmalen et al., 2024S-a)
- The online appendices (Rosmalen et al., 2024S-b):
  - Appendix 1: Prisma flow chart
  - Appendix 2: Frequency and categorization of the identified themes on toilet doors

RosmalenJ.
PlugI.
BalleringA.
 (2024S-a). All I want for Christmas...
[Research data]. PsychOpen. https://osf.io/gyqek/
10.32872/cpe.16159PMC1196056140177609

RosmalenJ.
PlugI.
BalleringA.
 (2024S-b). Supplementary materials to "All I want for Christmas is a loo: Visualizations of sex and gender on toilet doors"
[Online appendices]. PsychOpen. 10.23668/psycharchives.15751
PMC1196056140177609

## Data Availability

All raw visual data are available from OSF (see Rosmalen et al., 2024S-a).
